# Comparing Binding Modes of Analogous Fragments Using NMR in Fragment-Based Drug Design: Application to PRDX5

**DOI:** 10.1371/journal.pone.0102300

**Published:** 2014-07-15

**Authors:** Clémentine Aguirre, Tim ten Brink, Jean-François Guichou, Olivier Cala, Isabelle Krimm

**Affiliations:** 1 Institut des Sciences Analytiques, CNRS UMR 5280, Université de Lyon, Villeurbanne, France; 2 Centre de Biochimie Structurale, INSERM U1054, CNRS UMR5048, Université Montpellier 1 et 2, Montpellier, France; MRC National Institute for Medical Research, United Kingdom

## Abstract

Fragment-based drug design is one of the most promising approaches for discovering novel and potent inhibitors against therapeutic targets. The first step of the process consists of identifying fragments that bind the protein target. The determination of the fragment binding mode plays a major role in the selection of the fragment hits that will be processed into drug-like compounds. Comparing the binding modes of analogous fragments is a critical task, not only to identify specific interactions between the protein target and the fragment, but also to verify whether the binding mode is conserved or differs according to the fragment modification. While X-ray crystallography is the technique of choice, NMR methods are helpful when this fails. We show here how the ligand-observed saturation transfer difference (STD) experiment and the protein-observed ^15^N-HSQC experiment, two popular NMR screening experiments, can be used to compare the binding modes of analogous fragments. We discuss the application and limitations of these approaches based on STD-epitope mapping, chemical shift perturbation (CSP) calculation and comparative CSP sign analysis, using the human peroxiredoxin 5 as a protein model.

## Introduction

Fragment-based drug design (FBDD) has become a powerful approach for the generation of novel drugs against therapeutic targets [1], [2]. The first step of the FBDD process consists of identifying fragment-like compounds that interact with the protein, using biophysical techniques such as surface plasmon resonance, nuclear magnetic resonance, X-ray crystallography and mass spectrometry. The selection of fragments that will be further investigated and modified must be carefully done and depends on several criteria, including ligand efficiency (LE), lipophilic ligand efficiency (LLE), synthetic accessibility as well as specific protein recognition [1], [2]. One typically searches for fragments that bind the protein through a specific molecular recognition involving hydrogen bonds or charged interactions, rather than hydrophobic interactions that lead to non-specific recognition [3]. One way to identify specific protein-fragment interactions consists of comparing the binding modes of analogous fragments: fragments sharing key function moieties responsible for a specific intermolecular interaction should exhibit similar binding modes. Nevertheless, the addition of new chemical groups can induce a change of the binding mode, and one important task in FBDD is to check whether the main protein-ligand interactions are conserved or modified upon elaboration or modification of the fragment. Therefore, methods that allow us to rapidly compare the binding modes of analogous fragments are particularly valuable for the FBDD approach.

The binding modes of fragments are typically determined by X-ray crystallography [4], [5]. However, crystallography is not always successful due to crystallization difficulties or weak electron density for the ligand [6]. A main drawback of crystallography remains the frequency of false negatives for weak affinity fragments, in particular with the ligand-soaking approach. Alternatively high resolution NMR spectroscopy can be employed but routine methods based on filtered-NOESY experiments are usually time-consuming. Nevertheless, the NOE matching approach has been recently proposed to circumvent full protein resonance assignment [7], while the group of Siegal reported the successful use of sparse NOEs [8] and paramagnetic-induced pseudocontact shifts [9]. These methods can still be time-consuming when the objective is to compare the binding modes of a fragment series. In this report, we show how the ligand-observed saturation transfer difference (STD) experiment [10] and the protein-observed ^15^N-HSQC experiment, typically used for screening fragment libraries, can be adopted to compare the binding modes of analogous fragments, and for assessing whether the binding mode of the common motif is conserved upon binding. While the STD experiment allows a binding mode comparison through the epitope mapping effect observed on the measured peak intensities [11], the ^15^N-HSQC experiments can reveal ligand binding modes through the quantitative analysis of the chemical shift perturbations (CSPs) induced on the protein NMR spectrum upon ligand binding [12], [13]. Here, we assess the usefulness of these methods for small, weak affinity fragment-like compounds binding to the peroxiredoxin 5 protein, and we show that the combination of the two NMR experiments (STD and ^15^N-HSQC) including CSP calculation is required to assess the binding modes of fragments. We also show that assessment of the fragment binding modes is feasible through a comparative CSP analysis based on the experimental CSP signs only, as explained below. The two approaches presented here (calculation of CSP in combination with STD data, and comparative CSP sign analysis) are demonstrated to be efficient methods for comparing analogous fragments, and should have a direct impact in FBDD.

## Materials and Methods

### Protein Production and Purification

Protein production and purification was performed at *the Platform of IBCP-Lyon “Bioengineering of proteins”*. Human peroxiredoxin 5 PRDX5 was expressed as a 6xHis-tagged protein in *Escherichia coli* strain M15 using the pQE-30 expression vector. Cells were grown at 37°C in M9 minimal medium supplemented with thiamine and containing ^15^NH_4_Cl as the sole nitrogen source to produce uniformly ^15^N-labelled protein. Expression was induced with isopropyl β-D-1-thiogalactopyranoside for 4 h. Cells were then lysed in 20 mM imidazole, 20 mM sodium phosphate, 500 mM NaCl (pH = 7.4) supplemented with lysozyme and DNase by sonification and clarified by centrifugation. The 6xHis-tagged protein contained in the supernatant was purified using a His GraviTrap column (GE Healthcare) by Ni^2+^-affinity chromatography. The protein was eluted with 500 mM imidazole, 20 mM sodium phosphate and 500 mM NaCl (pH = 7.4). Eluted protein was then dialysed (3500 Da cutoff) against PBS buffer (pH = 7.4, NaCl 137 mM, KCl 2.7 mM, Na_2_HPO_4_ 10 mM, KH_2_PO_4_ 1.8 mM).

### STD Experiments

NMR samples for STD experiments [10], [15] were prepared with 20 µM PRDX5, 600 µM fragment in 0.5% DMSO-d_6_, 10% D_2_O (v/v), with PBS buffer (pH 7.4) and 1 mM 1,4-dithiothreitol (DTT). Fragments **1–5** used here are reported in Table 1. Standard 1D and STD NMR spectra were acquired at 20°C with a Varian Inova 600 MHz NMR spectrometer, equipped with a room temperature 5 mm triple-resonance inverse probe with z-axis field gradient. 1D and STD experiments were performed using identical experimental conditions (spin lock, interscan delays), and parameters for the STD experiments (saturation frequency and saturation time) were identical for all samples. Selective saturation of the protein NMR spectrum was achieved with the decoupler offset 3000 Hz upfield from the carrier frequency, and non-saturation control was performed at 15000 Hz downfield. The number of scans was set to 800 for STD experiments and 400 for the 1D. STD signals were measured for protons in the aromatic region only. For each fragment NMR signal the ratio R between the intensities of the STD signal and the 1D signal was calculated (R = (I_STD_/I_1D_) *100). STD spectra were normalised by setting the largest observed ratio to 100%.

**Table 1:** Affinities of fragments 1 to 5 to the PRDX5 protein.

Fragments	Name	MW (g/mol)	K_D_ (µM)*	LE**
**1**	catechol	110.11	1500±500	0.49
**2**	4-methylcatechol	124.14	330±40	0.54
**3**	4-tert-butyl-catechol	166.22	50±20	0.54
**4**	1–1′-biphenyl-3,4-diol	186.21	150±20	0.38
**5**	2,3 dihydroxy-biphenyl	186.21	390±50	0.34

*Average values ± standard error of the mean.

**LE = ΔG/(number of heavy atoms).

### ^15^N-HSQC Experiments

NMR samples contained 200 µM uniformly ^15^N-labeled protein, 5 mM DTT, and ligand concentration was varied between 0–2 mM. 2D ^15^N−HSQC spectra were acquired at 28°C, using 64 t_1_ increments. A control 1D ^1^H spectrum was recorded prior to each ^15^N−HSQC experiment to assess the purity and stability of the fragments. Solutions at maximal fragment concentration were checked for alteration of the sample pH to prevent confounding sources of CSP. All NMR spectra were processed using Varian VnmrJ and NMRPipe [16] and analysed using NMRView [17] and Sparky [18].

### CSP Measurements

For a given ^15^N−HSQC cross peak the proton and nitrogen CSPs (CSP_H_ and CSP_N_ respectively) induced by fragment binding were defined as the difference between the corresponding chemical shifts in the bound and the free states:
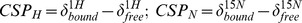



### K_D_ Measurement

All of the complexes between PRDX5 and fragments **1–5** exhibited behaviour consistent with being in the fast exchange regime. The respective dissociation constants were obtained from the concentration dependence of the combined CSP (*CSP*_(H+N)_)
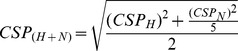
by fitting a plot of the *P*_0_/*L*_0_ ratio against *CSP*_(H+N)_ using an in-house fitting procedure [19] according to:



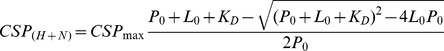
where *P*_0_ and *L*_0_ are the total protein and the ligand concentration, respectively. *CSP_max_* is the maximum chemical shift change at saturation, obtained by the fitting procedure. The final K_D_ was obtained by averaging K_D_ values from individual fits for a subset of cross peaks that showed the largest CSP upon fragment binding.

### X-Ray Crystallography

Crystals were grown by hanging drop vapour diffusion at 18°C by mixing 1 µl of protein solution (25 mg/ml) with 1 µl of well solution composed of 22% PEG3350, 0.1 M sodium citrate buffer (pH 5.3), 0.2 M potassium sodium tartrate, 5 mM 1,4-dithiothreitol, 0.02% (w/v) sodium azide. Crystals appeared after one day with typical dimensions 0.3–0.5 mm. Soaking experiments were performed for fragments **1**, **2** and **3** by adding 0.2 µl of a 100 mM solution in DMSO before flash-cooling at 100 K in liquid nitrogen. Fragment **4** was co-crystallized with the protein and directly flash-cooled in liquid nitrogen. The data were collected on beam-lines ID23-1 and ID14-4 at the ESRF (Grenoble, France). All measurements were indexed and integrated using iMOSFLM program [20] and merged with the SCALA program. Statistics for data collection and processing are given in Table S1. The solution of the crystal structures were obtained by the molecular replacement method using the program MOLREP of the CCP4 [21] suite and the structure 1HD2 as a model [22]. The structures were refined using the program REFMAC5 of the CCP4 suite. The structures have been deposited at the RCSB Protein Databank (PDB codes 4K7I, 4K7N, 4K7O and 4MMM).

### Docking

All docking computations were performed with AutoDock4.2.3 [23]. 200 independent runs were conducted for each fragment using the Genetic Algorithm with standard settings. Structure PDB entry 3MNG containing PRDX5 in interaction with DTT was used as the 3D template for the docking, as no apo-structure is available. The DTT coordinates were removed from the crystal structure. Protein and fragment structures were prepared with AutoDock Tools. The standard AutoDock-Potential scoring function was used.

### CSP Calculation

Ligand-dependent CSPs were corrected for any CSP effect induced by DMSO. Instances of measured CSP_H_ smaller than 0.02 ppm were set to 0. The CSP calculation was based on the ring current effect due to aromatic rings [24] and an electric field term [25] was added for partial charges. No other contribution to the ligand-induced CSP was included in the calculations due to the lack of appropriate models [26]–[28]. The ring current effect for *CSP*_H_ was calculated using the Haigh-Maillon semi-classical model [24]:
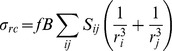
Here, *f* is the ring-specific intensity factor (e.g. 1.00 for benzene type ring), *B* is the target nucleus factor (*B* = 7.06*10^−6^ Å for amide protons [29]). Other values for *f* and *B* can be found in reference [30]. The sum is calculated over pairs of bonded ring atoms with *i j* ∈ {1,2;2,3;3,4;4,5;5,6;6,1}. *r_i_* and *r_j_* correspond to the distances from the ring atoms *i* and *j* to the amide proton of the protein, respectively. *S_ij_* is the (algebraic signed) area of the triangle formed by atom *i* and *j* and the target amide proton projected onto the plane of the aromatic ring.

For charges, an electric field model [25] was employed:
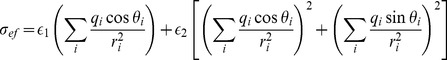
ε1 = −2.0*10−12esu and ε2 = −1.0*10−18esu were used [25]. The sum is over all ligand atoms, ri is the distance between atom i and the amide proton, qi is the partial charge of atom i and θi is the angle between the NH vector of the target amide group and the HN-i vector. The electric field effect due to each of the fragments 1–5 was nearly negligible.

To evaluate the agreement between the predicted and measured *CSP*_H_ the *P_score_*, the normalized version of the *Q_score_* used by McCoy and Wyss [12], was used:
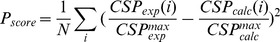
Here *N* is the number of residues, *CSP_exp_*(*i*) is the experimental CSP value for residue *i*, *CSP_calc_*(*i*) is the calculated CSP value for residue *i,* and 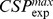
and 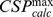
are the largest (irrespective of sign) observed and calculated CSP values over all residues, respectively. A low *P_score_* indicates that the docking solution is in good agreement with the experimentally observed CSPs.

## Results

To investigate the applicability of the two most popular NMR screening paradigms (STD and ^15^N-HSQC) for assessing the binding modes of analogous fragments, we chose as a protein model the human peroxiredoxin 5 enzyme (PRDX5), one of the six peroxiredoxin enzymes involved in post-ischemic inflammation in the brain [14], [31], [32]. As verified by molecular dynamics (MD) simulation, the protein does not undergo substantial conformational change upon ligand binding (data not shown), which makes it an appropriate model here, since the experimental CSPs contain mainly direct contributions of the ligand binding. In this report, we examine and compare the binding modes of fragments **1**–**5** that each contains a catechol moiety (Table 1). As reported in the literature, catechol groups can behave as pan assay interference compounds (PAINS) in screening experiments [33], leading to false-positive hits. To ensure that the catechol-containing compounds **1**–**5** reversibly interact with the PRDX5 protein and that the binding signals are not artefacts, the redox and oligomeric states of the protein were carefully checked by NMR, both in the free and fragment-bound forms. The protein NMR spectra showed that the redox state and the oligomeric state of the protein were not modified upon ligand addition.

### STD experiments

The binding of the PRDX5 ligands **1**–**5** was investigated using STD experiments. If a ligand specifically binds the protein with a single binding mode, ligand protons that are buried into the protein can be distinguished from solvent exposed protons: STD signals of solvent exposed protons are weak compared to those of buried interfacial protons, translating into the so-called epitope mapping effect [10], [11]. By contrast, if a ligand binds to the protein through hydrophobic non-specific interactions, or displays multiple binding modes, no epitope mapping should be observed in the STD spectrum. Nevertheless, if the T_1_ relaxation times of individual ligand protons are significantly different, STD experiments may not give an quantitatively reliable epitope map [34]. Therefore, comparison of STD signals for different ligand compounds must be carried out carefully and must involve similar protons. Here, only the STD signals of aromatic protons of the fragments were analysed.

**Figure 1:**
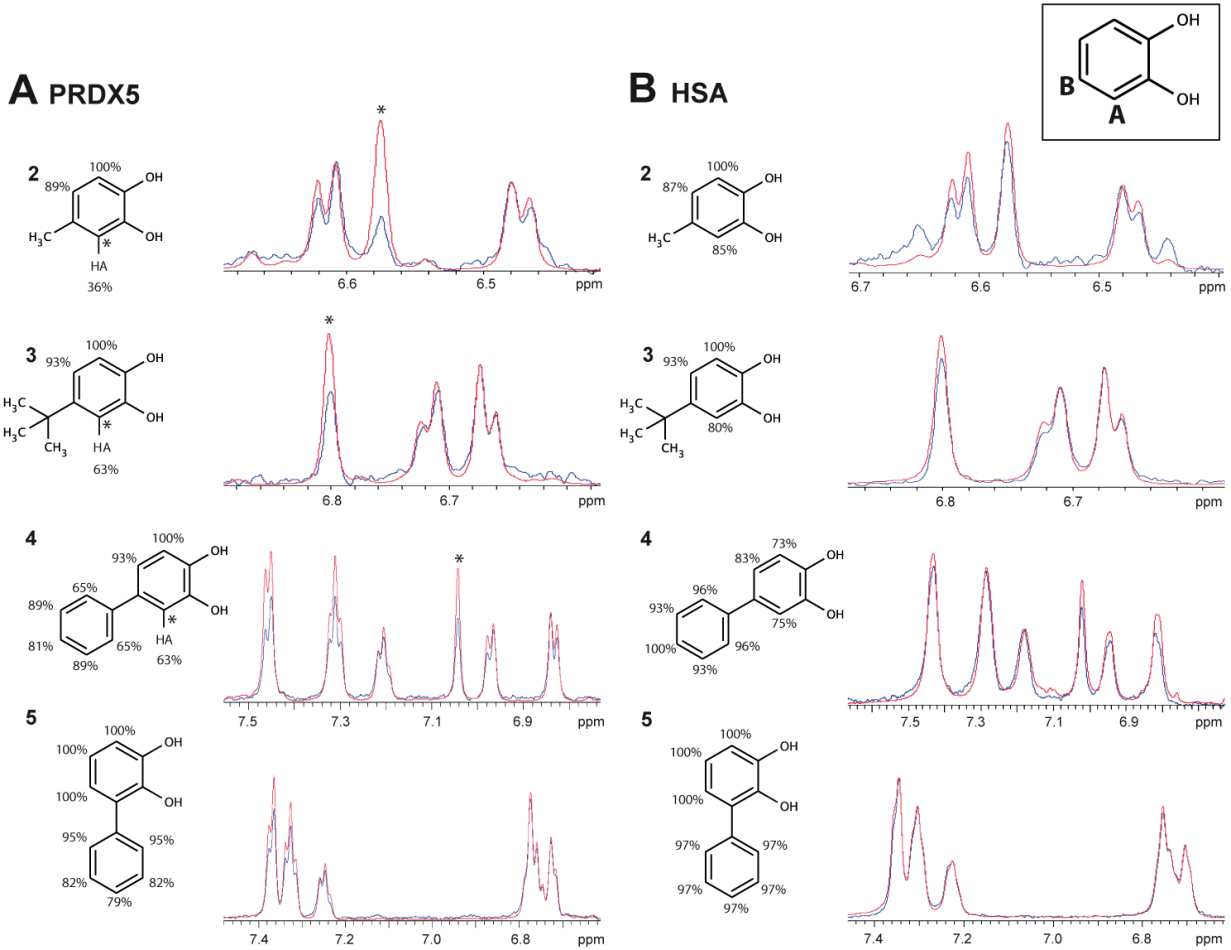
STD investigation of fragment binding to PRDX5. 1D ^1^H NMR spectra (in red) are superimposed to STD NMR spectra (in blue). (A) NMR experiments in the presence of PRDX5, (B) NMR experiments in the presence of HSA. The relative STD effects (R ratio, see Material and Methods) measured for the aromatic protons are indicated. The proton in position HA, labelled with an asterisk (*), exhibits a weak STD effect for fragments 2, 3 and 4, upon binding to PRDX5, indicating that the proton HA is solvent exposed. This effect is not observed in the presence of HSA. STD spectra were scaled by setting the largest ratio to 100%. Positions A and B are displayed on the catechol (top right corner).

STD spectra of compounds **2**–**5** recorded in the presence of PRDX5 are displayed in Figure 1, and the STD factors (calculated as R, see Material and Methods) are indicated. Since only one NMR peak is observed for fragment **1**, no STD factor was calculated. For fragments **2**, **3** and **4**, the relative intensity of the HA proton resonance differs in the STD spectrum compared to that observed in the corresponding 1D spectrum (Figure 1A). These observations indicate that the fragments **2**, **3** and **4** bind to PRDX5 with a particular orientation of the catechol moiety, where the proton HA is exposed to the solvent, and other protons are buried. The STD spectrum for compound **5** is less informative, but still suggests that the catechol moiety is the part of the ligand that is buried upon PRDX5 binding, since protons of the catechol moiety exhibit slightly higher STD factors than the protons of the second aromatic ring.

For comparison, STD experiments were recorded in similar conditions in the presence of human serum albumin (HSA) in place of PRDX5. As shown in Figure 1B, the epitope mapping effect detected with PRDX5 is not observed in this case, confirming the absence of a preferred binding mode of compounds **2**–**5** to serum albumin, which suggests that interactions are mostly driven by hydrophobic interactions.

In conclusion, the STD experiments suggest that the catechol moiety of compounds 2–4 specifically bind to PRDX5 and adopt a similar orientation upon binding to the protein, with their HA proton exposed to the solvent. Nevertheless, additional information inferred from HSQC experiments are required to ensure that the catechol moieties have the same orientation in the complexes.

### HSQC experiments and K_D_ Measurements

**Figure 2:**
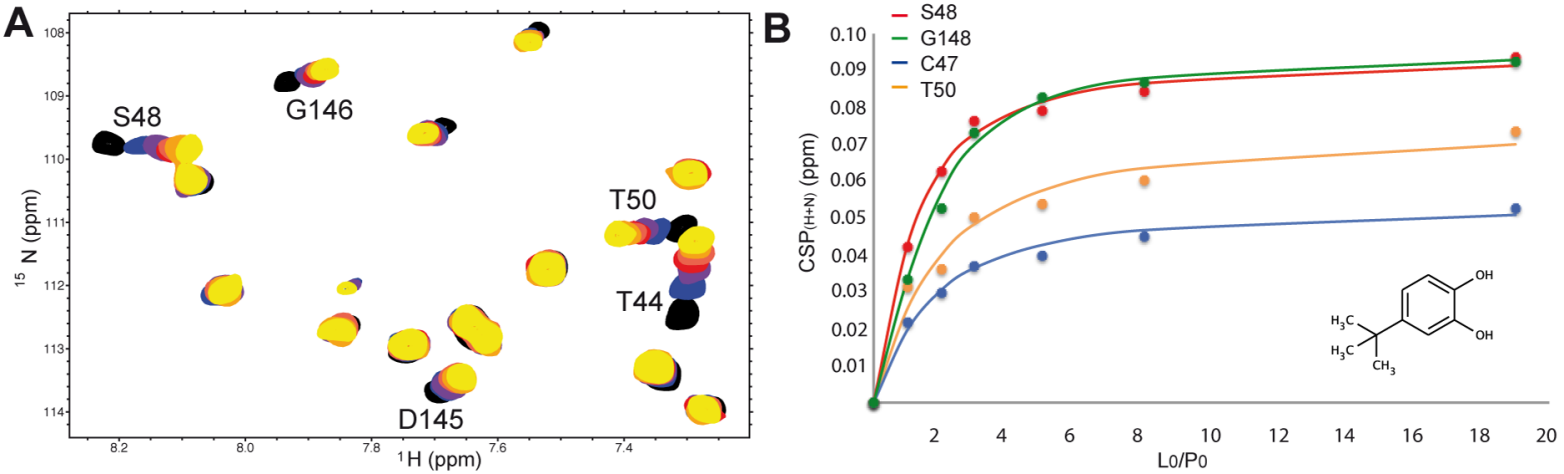
Chemical shift perturbations and affinity measurement for the PRDX5-fragment 3 complex. (A) Section of the ^15^N-HSQC spectrum, with the superimposition of the free protein spectrum (black) and spectra with increasing fragment concentration (110 µM blue, 220 µM violet, 330 µM red, 550 µM light red, 880 µM orange, and 2 mM yellow). (B) Titration curves obtained from ^15^N-HSQC spectra. Combined CSP_(H+N)_ were measured for each fragment concentration. Curves obtained for residues S48, G148, C47, and T50 are shown.

^15^N-HSQC experiments recorded for fragments **1**–**5** show that the CSPs observed upon ligand binding involve residues located in the protein active site (residues 42, 44, 46–51) and in protein regions around the active site (residues 75–80, 112–114, 116–119,121–123, 143, 145–148, 151–152). This confirms that all fragments bind to the enzyme active site. Dissociation constants for fragments **1**–**5** were measured using ^15^N-HSQC experiments recorded with ligand concentration ranging from 0 to 2 mM with 200 µM PRDX5 (Figure 2). As reported in Table 1, affinities vary from 50 µM for compound **3** to 1500 µM for fragment **1**, leading to LEs that range from 0.34 (fragment **5**) to 0.54 (fragments **2** and **3**). These measurements indicate that the addition of a tert-butyl group at position B (Figure 1) is an efficient modification. By contrast, addition of a phenyl group at position A induces a loss of affinity. Experimental CSPs measured at 2 mM ligand concentration were then compared to calculated CSPs, in order to better understand and assess the ligand binding modes.

### CSP calculation

Calculation of protein ^1^H CSPs observed upon ligand binding has been reported in previous papers for resolving the 3D structure of protein-ligand complexes [12], [13], [26], [35], but this methodology is not routinely used. Because such an approach could be of great interest in FBDD, we have tested the method here for the PRDX5-fragment complexes. The process requires the generation of virtual positions of the ligand in the protein 3D structure by computational docking, followed by the prediction of the expected CSPs for protein protons for each ligand pose. The calculation of ^15^N CSP has not been described, due to the lack of suitable empirical models [19], [36]. Calculation of ^1^H CSP is mainly based on the contributions provided by the ring current effect induced by aromatic rings, on the electric field effect due to charges and partial charges, and on anisotropic effect due to double bonds such as carbonyl groups [25], [37]. The binding mode of the ligand is taken as that which exhibits the best agreement between calculated and experimental protein ^1^H (usually the amide protons) CSPs.

**Figure 3:**
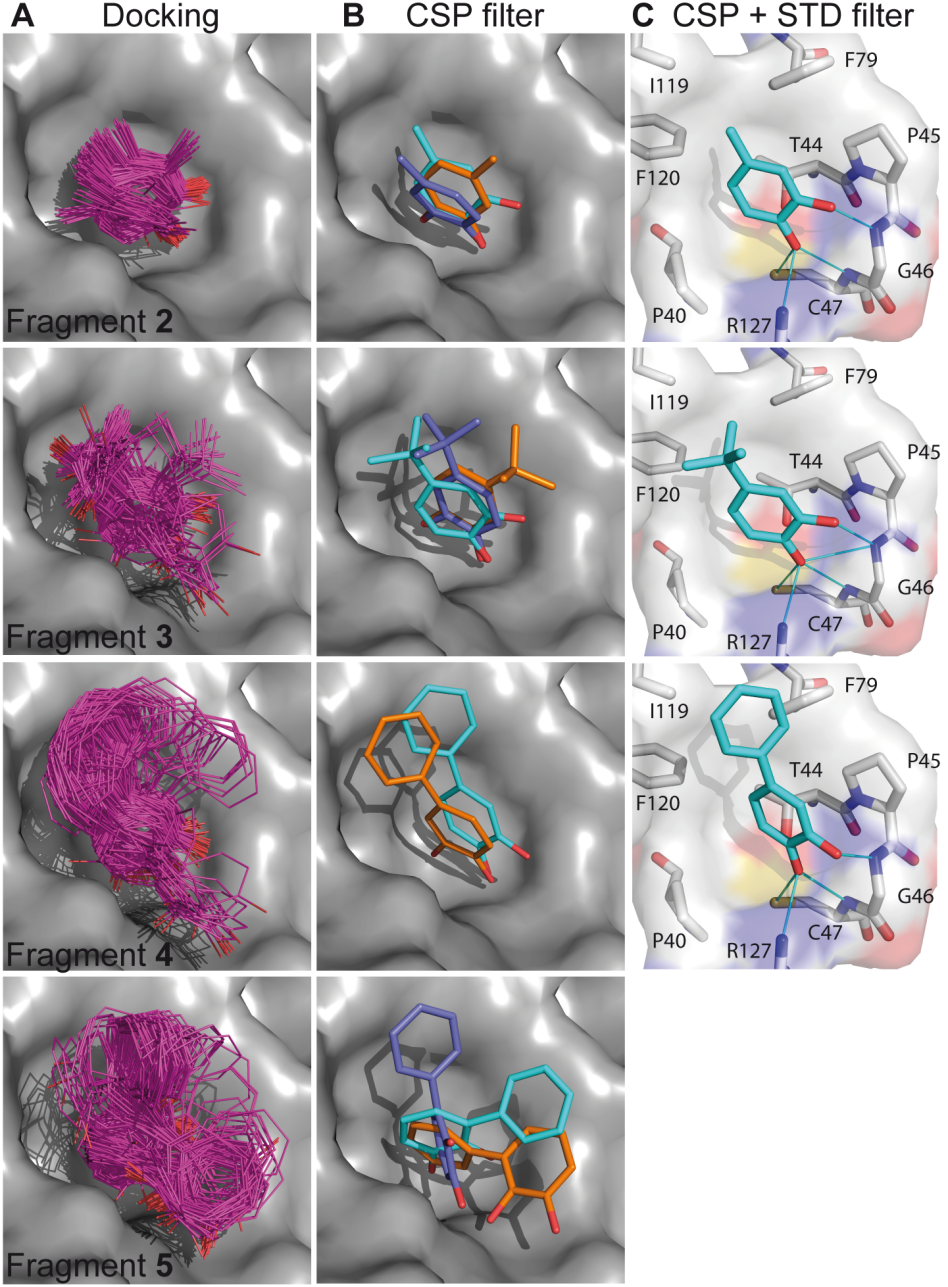
Binding modes of the fragments determined by CSP calculation and STD. (A) 200 ligand orientations generated by docking (B) CSP filter: binding modes of the fragments obtained by filtering the positions according to their agreement between experimental and calculated CSP. (C) Combined CSP and STD filter: binding modes of the fragments in agreement with both CSP calculation and STD data. Hydrogen bonds, identified using Ligplot + [41], are displayed in blue lines.

PRDX5-fragment complex structures were generated using AutoDock software [23], and the CSPs were calculated for each ligand position, as described in the experimental section. The fragment orientations exhibiting the best agreement between the experimental and calculated CSPs are selected using the *P_score_* value. Starting from 200 ligand orientations for each fragment (Figure 3A and Table S2), the CSP filter selected ligand positions exhibiting roughly the same orientation of the catechol group for fragments **2**–**4** (Figure 3B), indicating that the CSPs support a conserved binding mode for these compounds. Regarding fragment **5**, the catechol moiety does not superimpose well on those of fragments **2**–**4** for any of the selected binding modes, suggesting that the addition of a phenyl group at position A induces a binding mode change that translates into a different orientation of the catechol ring (Figure 3B). The results also illustrate the limits of the CSP calculation method for resolving the binding modes of the fragments, since multiple binding modes are compatible with the CSP data. As shown in Figure 3B, three binding modes are selected for fragments **2**, **3** and **5**, and two binding modes are in agreement with the CSP calculation for fragment **4**. The main limitation is that the CSP calculation does not consider the influence of the substituents such as methyl and tert-butyl groups, nor the hydroxyl functions. Additional issues are observed when experimental CSP magnitudes are weak, as exemplified for fragment **1** (Figure S1). Nevertheless, despite the limitation of the method, the CSP calculation shows that the catechol ring orientation is conserved for fragments **2**–**4** but not for fragment **5**.

### Combination of STD and CSP calculation

We have then used the STD data to further refine the binding modes of the fragments and find the positions of the substituents that were not clearly defined by CSP calculation. According to the STD experiments, the HA proton of fragments **2**–**4** is directed towards the solvent, and not buried in the protein surface (Figure 1A). As a consequence, some of the binding modes selected by the CSP calculation can be eliminated by comparing the solvent accessibility of the catechol protons in the various protein-fragment complex models. For fragments **2**–**4**, one unique binding mode is obtained from the combination of STD and CSP data (Figure 3C). The binding mode appears to be determined by the hydroxyl groups that form hydrogen bonds with the protein backbone amide of residues G46 and C47. In these NMR-derived models, the substituents (methyl, tert-butyl and phenyl groups) are located near the 113–125 loop connecting the α-helix (residues 104–110) to the β-strand (residues 127–133), forming hydrophobic interactions with residues L116, I119 and F120, while the catechol moiety resides in a common position in the protein active site (Figure 3C). For fragment **5**, the STD spectrum is not helpful to refine the ligand orientations selected by the CSP calculation, thus the binding mode for this fragment cannot be further elucidated by this approach.

### Comparative CSP sign analysis

**Figure 4:**
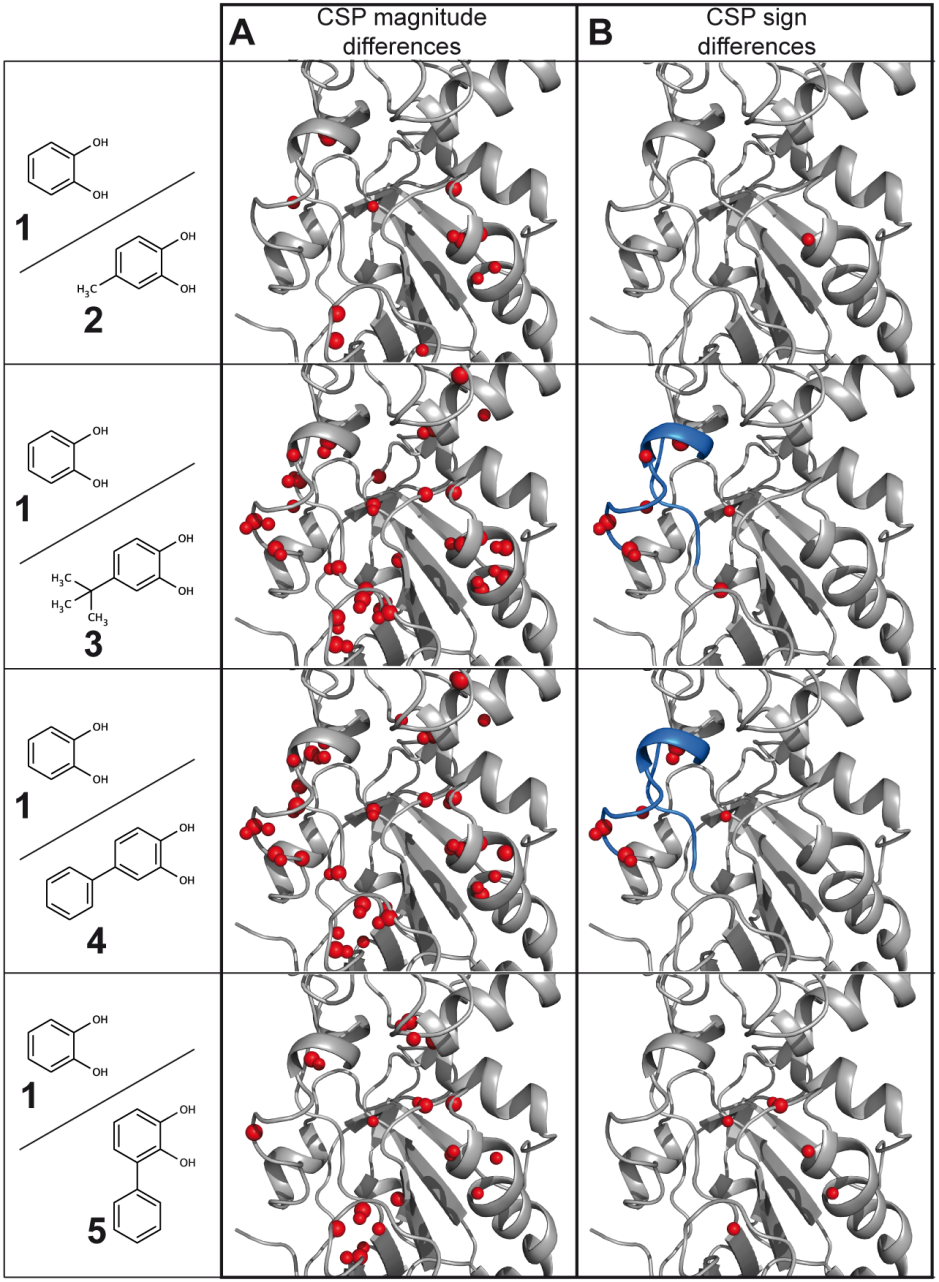
Comparative experimental CSP analysis. CSP observed on PRDX5 spectra when bound to fragments 2, 3, 4 and 5 are compared to CSP observed in the presence of fragment 1. Residues displaying CSP magnitude (A) or CSP sign (B) differences are displayed with small or large red spheres, for protons and nitrogens, respectively. (A) Comparison of the CSP magnitudes. Spheres are displayed in the case of the absolute CSP differences are larger than 0.02 ppm for protons and 0.1 ppm for nitrogens. (B) Comparison of the CSP signs. Spheres indicate experimental CSP signs differences. The loop 113–125 is coloured in blue.

In addition to CSP calculation, we also assessed whether the relative binding modes of analogous fragments could be inferred from the comparison of the corresponding experimental CSPs. Comparative CSP analysis was previously proposed to localise the region of the binding site that is proximal to the part of the ligand that differ from one ligand to another within a series of analogues [19]. The approach published by Fesik and co-workers involves the comparison of chemical shift changes for the protein induced by a series of closely related ligands [38]. The method is particularly useful for large ligands, but seems inappropriate when dealing with a series of fragments with strong differences in the CSP magnitudes, since CSP differences in this case will appear for all residues of the protein exhibiting CSPs, and not only for the residues in proximity to parts of the ligands that differ from one ligand to another (Figure 4). For example here, maximal proton CSP magnitudes are 0.05 ppm for fragment **1** and 0.16 ppm for fragment **4**. As illustrated in Figure 4A, comparison of CSPs induced by fragment **1** to CSPs induced by fragments **2–5** highlight differences located all around the binding site, preventing any conclusion regarding the relative binding modes of the fragments. In another approach previously reported by Riedinger et *al*., both the signs and the magnitudes of the CSPs are taken into account [39]. Nevertheless, the method does not allow the comparison of the ligand binding modes for fragments exhibiting very diverse CSP magnitudes.

To overcome the CSP magnitude issue, we propose to compare only the experimental CSP signs without any CSP calculation. To do so, the experimental proton and nitrogen CSP profiles along the protein sequence are plotted for each fragment, as shown in Figure S2. CSP values are positive if the atom is affected by a deshielding effect, and negative in case of a shielding effect. While CSP profiles induced by the fragments exhibit obvious differences when comparing the CSP signs (Figure S2), no differences are observed between the CSP profiles when the combined CSP values, which only contain absolute magnitudes, are used (Figure S3). As shown in Figure 4B, only one residue exhibits a CSP sign difference when CSPs induced by fragments **1** and **2** are compared, while comparing CSPs for fragment **3** to fragment **1** highlights sign differences located in the vicinity of the 113–125 loop. Similarly, comparing CSPs for fragment **4** to fragment **1** highlights sign differences in the 113–125 loop. Regarding fragment **5**, differences with fragment **1** are observed in various regions of the binding site, with opposite CSP sign observed for residues A42, G46, H51, V75 and G148 (Figure 4B).

The comparative CSP analysis based only on the CSP signs allows one to draw some conclusions regarding the fragment binding modes. When the CSPs induced by fragments **1** and **2** are compared, all residues but G46 exhibit identical CSP signs (Figure 4B), demonstrating that the fragments share a similar binding mode with a similar ring orientation. A different catechol orientation would generate CSP sign differences for protein protons located all around the catechol group (Figure S4). For both fragments **3** and **4**, the fact that the CSP sign differences are observed in a localised region (113–125 loop) indicates that their bulky substituents are positioned towards this region and that their catechol moieties and fragment **1** have the same orientation, as further discussed below. For fragment **5**, CSP sign differences are located at various regions of the protein, including active site residues (Figure 4B), suggesting that the binding mode is different. This finding is corroborated by CSP calculation performed for PRDX5-fragment **5** docking model in which the catechol moiety of fragment **5** and fragments **2–4** are superimposed. According to the CSP calculation, the sign differences observed in the active site region CSPs must be assigned to the influence of the catechol moiety (and not to the second ring in fragment **5**), therefore demonstrating that the binding mode of the catechol moiety is modified for fragment **5**.

### X-Ray crystal structures

**Figure 5:**
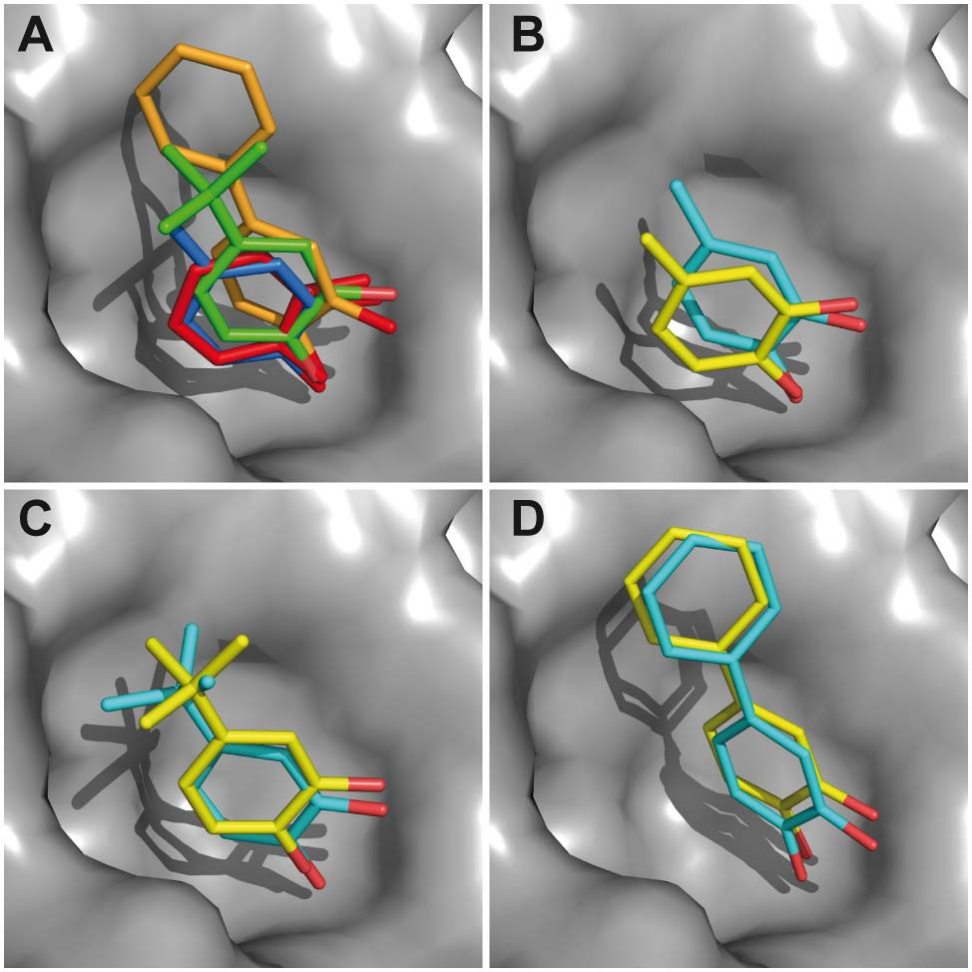
Comparison of NMR and X-ray protein-fragment structures. (A) Superposition of the X-ray structures of the complexes for fragments 1 (red), 2 (blue), 3 (green) and 4 (orange). (B) The NMR-derived binding modes (cyan) are compared to the X-Ray structures (yellow) for fragments 2 (B), 3 (C) and 4 (D). Fragment positions were extracted from the solved X-Ray structure and are displayed in the 3MNG protein structure (used for docking).

To further demonstrate that the comparative CSP-sign analysis (Figure 4B) is robust and to assess the orientation of the fragments obtained with the combination of the CSP calculation and STD data (Figure 3C) with X-ray structures, we have solved the corresponding PRDX5-ligand structures by X-ray crystallography. Complexes of fragments **1**, **2** and **3** were obtained by crystal soaking, while co-crystallisation was required for fragment **4**. The electronic density maps of the fragments are shown in Figure S5. Unfortunately, no crystal structure could be obtained for fragment **5**, likely due to its weak affinity and relatively poor solubility, which prevent a sufficient binding site occupation. This weak binding site occupation is no problematic for the observation of the binding event by NMR. As shown in Figure 5, the binding modes predicted from the NMR data are quite similar to those observed in the crystal structures, demonstrating that the combination of CSP calculation and STD data was an efficient way to derive the binding mode of the fragments. The atomic coordinate RMSD between NMR-derived and X-Ray structures ranged from 1.1 Å to 1.5 Å. The crystallographic results also show that the catechol moieties in ligands **1–4** directly superimpose, in line with conclusions from the comparative CSP sign analysis or from the CSP calculation approach for fragments **2–4**.

## Discussion

We show here that NMR ligand screening paradigms commonly used to identify fragment hits against protein targets, the STD and HSQC experiments, can be used semi-quantitatively to compare the binding modes of analogous fragments. The comparison of fragment binding modes is an important task for assessing the binding specificity and to highlight key interactions involved in the protein-fragment recognition. In addition, it is crucial in the FBDD process to verify whether the binding mode of fragments is conserved upon chemical structure elaboration.

In the case presented here, the catechol group represents the minimal motif shown to bind the PRDX5 protein. Questions we aimed to address using NMR are the following: does the catechol moiety specifically bind to PRDX5 (through interactions involving the hydroxyl functions)? Is the pose of the catechol motif maintained over the fragment series **1–5**, or does the addition of hydrophobic groups at positions A and B alter the binding mode?

To compare and characterize the binding modes of analogous fragments, we used STD and HSQC experiments. The STD experiment is not limited by the protein size, and the STD effect is all the more efficient if the molecular weight of the protein binding target is large. STD intensities for the ligand protons are related to the proximity of ligand and protein protons, and therefore indirectly highlight solvent exposed protons of the bound ligand [11]. This STD group epitope mapping analysis can be done only for protons that have a similar T_1_ relaxation time. For fragments **2–4**, the proton HA of the catechol group was shown to be solvent exposed in the protein-fragment complexes, by comparison with the other aromatic protons that are buried in contact with the protein surface. This is fully confirmed by the complex crystal structures. The STD data suggest that the binding mode of the catechol moiety is similar for the three fragments, showing that meaningful conclusions can be drawn from STD experiments regarding relative binding modes. In addition, comparison of the STD spectra observed in the presence of the protein target or human serum albumin is an efficient way for comparing binding modes. Whilst it is likely that a fragment will also bind serum albumin, it is very unlikely that a similar binding mode will be observed.

Regarding protein-observed experiments, CSPs measured with HSQC experiments can be exploited for structural information, by selecting computational models in which the ligand is positioned with a ring orientation displaying the best agreement between experimental and calculated CSPs [12], [13], [26]. By contrast with the use of STD data, the CSP calculation requires knowledge of the protein 3D structure. The main limitation of the CSP calculation approach is that hydrogen bond effects are not simulated, since adequate models to predict the effect on chemical shifts are not available yet. One consequence can be that ring orientations that compensate for the hydrogen bond effects are erroneously selected, leading to binding modes that are not the correct structures [26]. The risk of generating wrong orientations is more pronounced when experimental CSP magnitudes are weak, as observed for fragment **1** (see Figure S1). In addition, ligand-dependent conformational rearrangement of the protein can prevent the use of CSP calculation for binding mode assessment, if the conformational changes alone induce large CSPs. Such structural events are evidenced by the disagreement observed between experimental CSP and CSP calculated for the ligand orientations [40]. While STD measurements will still give meaningful results for the comparison of the binding modes of analogous ligands, the CSP filter might fail to select the correct ligand position. With regard to CSP sign analysis, the method might be useful for the detection of differential conformational changes induced by each of a series of ligands. In this respect, PRDX5 is a prime example due to its relatively rigid binding site. Owing to the limitations of CSP calculation, additional experimental constraints may be required to accurately model the complex structure. We show here that CSP calculation can usefully be combined with additional data such as STD intensities to identify the ligand-binding mode in the case of PRDX5-fragment complexes. As shown in Figure 5, the NMR-derived binding modes based on a combination of STD and CSP data are close to those observed in the corresponding X-ray crystal structures. Despite the limitations of the CSP calculation approach, it nevertheless proved useful to show that the addition of a phenyl substituent in position A causes a modification of the binding mode (Figure 3B). CSP calculation can be performed for fragments regardless of their affinities, since the experimental CSP are normalised for the *P_score_* quantification (see Materials and Methods). Therefore, the ranking of the ligand orientations is not altered by the complex concentration. Importantly, while CSP calculation can be performed for non-related fragments, the comparative CSP analysis should be performed for closely related fragments only.

**Figure 6:**
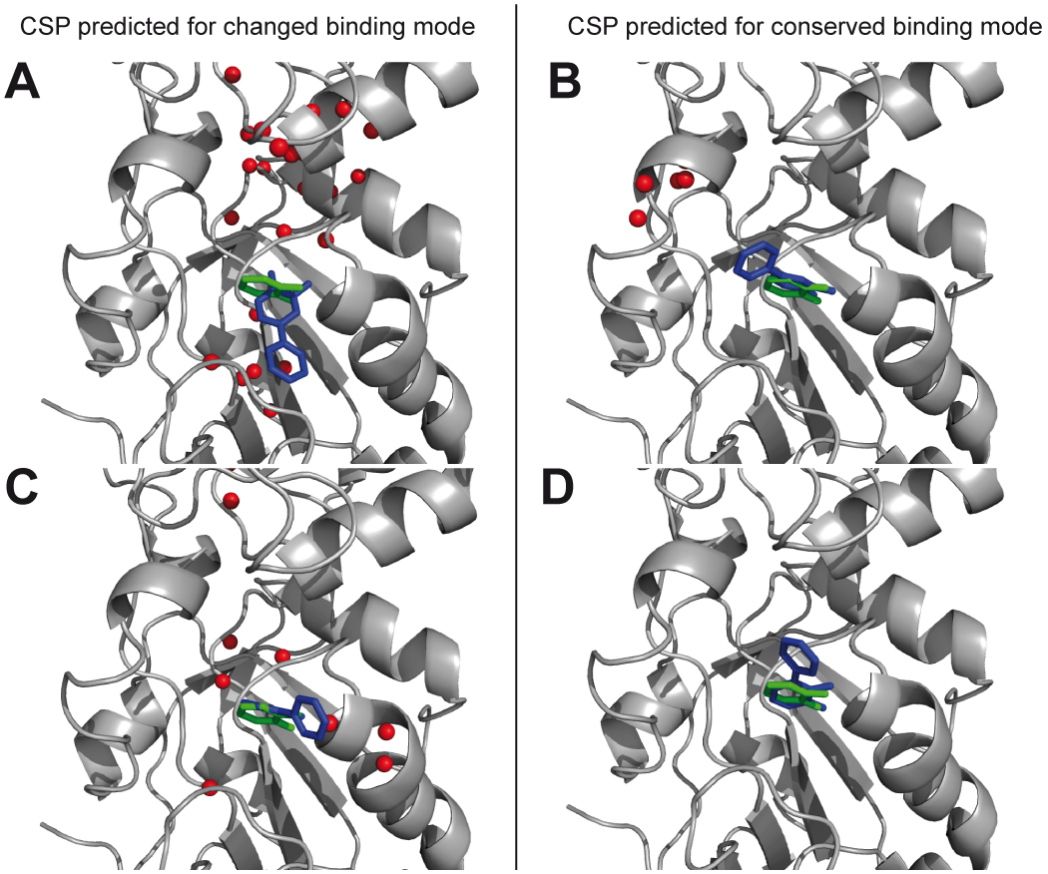
Calculated CSP sign differences between binding modes of analogous fragments. Comparative CSP sign analysis in case of binding mode conservation (left) or binding mode change (right) of the catechol group for the fragment pairs 1/4 (A) and 1/5 (B) demonstrating a conserved binding mode between fragments 1 and 4 and a binding mode modification between fragments 1 and 5. (A) CSP signs differences are expected for residues located in the loop 113–125 in case of superimposition of the catechol moieties of fragments 1 (green) and 4 (blue) (in agreement with experimental CSP data), while CSP signs differences are expected in numerous protein regions in case of a change of the catechol orientation. (B) No CSP signs differences are expected in case of superimposition of the catechol moieties of fragment 1 (green) and 5 (blue), while CSP signs differences are expected in the active site region in case of a change of the catechol orientation (in agreement with experimental CSP data). Spheres are displayed only if one of the two compared fragment protons displayed calculated CSPs larger than 0.02 ppm.

As illustrated in Figure 4, the comparison of analogous fragment CSPs should take only CSP signs into account to counteract the CSP magnitude effects observed with ligands exhibiting significant affinity differences. Importantly, the CSP sign is not affected by the ligand affinity. One important consequence is that it is possible to compare ligands without any assumption of their affinities. This can be useful for the efficient comparison of complexes using only a single HSQC spectrum recorded at ligand concentrations that differ from one fragment to another, depending on the solubility limit of the ligand. Even when the CSP magnitudes are similar, comparing the CSP sign is the best way for highlighting binding differences. For example, the CSP sign of fragments **1** and **5** are clearly different in the active site region (Figure S2), while the commonly-adopted CSP analysis, based on the absolute magnitudes of proton and nitrogen CSP, would not highlight binding mode differences between the two fragments (Figure S3). The robustness of the comparative CSP sign analysis is further illustrated in Figure 6, where predicted CSP sign differences expected between fragments **1** and **4** (Figure 6A), and fragments **1** and **5** (Figure 6B), are displayed for conserved catechol binding mode, and in the case of a binding mode variation. A good agreement is observed with experimental data shown in Figure 4B, confirming that fragments **1** and **4** share a similar binding mode, whilst the lack of agreement for fragments **1** and **5** is consistent with a difference in binding modes.

Regarding fragment **5**, three binding modes selected by the CSP calculation (Figure 3B) were compared to the binding mode of fragment **1** using the comparative CSP sign analysis. The calculations suggest that the orientation of fragment **5** displayed in Figure 3B is the best NMR-model (Figure S6). This illustrates that CSP calculation may be combined with comparative CSP sign analysis to further analyse the binding modes of fragments by NMR.

## Conclusion

The NMR-based comparison of the binding modes of the fragments **1–5** lead to three conclusions (1) fragments **1**, **2**, **3** and **4** have a conserved binding mode in PRDX5 complexes with a common orientation of the catechol moiety, suggesting that the catechol group alone recognizes the protein active site through hydrogen bonds involving the backbone H-bonding groups of residues G46 and C47, (2) the hydrophobic catechol substituents for fragments **3** and **4** point towards the loop 113–125, (3) addition of a phenyl group at position A (fragment **5**) induces reorientation of the catechol framework in the enzyme active site, likely to minimize the solvent exposure of the second ring, which leads to an affinity decrease. By contrast, addition of hydrophobic groups at position B does not modify the catechol binding mode, and leads to higher affinity and even to a ligand efficiency (LE) increase in case of the methyl and tert-butyl groups. This report exemplifies how NMR screening experiments can be used in a semi-quantitative manner to further characterize the binding properties of fragments, through the binding mode comparison of analogous fragments.

## Supporting Information

Figure S1 **CSP-driven binding mode for fragment 1.** The NMR structure is shown in cyan, the X-Ray structure is coloured in yellow, and a docking position close to the X-Ray structure is shown in green. Fragment position was extracted from the solved X-Ray structure and is displayed in the 3MNG protein structure (used for docking). As shown in Figure S1, the binding mode selected by the CSP calculation for compound 1 is quite different form the X-Ray structure (rmsd 2.69 Å). This result arises form the Pscore filter that identifies as the best position the orientation displayed in cyan and not the orientation coloured in green (rmsd 0.36 Å to the X-Ray structure). Here, additional NMR data would be required to select the structure displayed in green. This false positive result highlights limitation of the CSP calculation, similarly to the function scoring issues reported for fragment docking. The limitation of the CSP calculation is likely to increase for cases where experimental CSPs are small and/or measured for a small set of protein residues. For example here, a smaller number of CSPs were used for fragment 1 as compared to the others compounds.
(TIF)

Figure S2 **CSP profiles for fragments 1–5, observed for protein protons (left) and protein nitrogen atoms (right).** CSP profiles are superimposed for fragments 1 and 2, showing few differences (A, B), fragments 1, 3, 4, showing differences for the loop 113–125 (C, D) and fragments 1 and 5, showing differences in the active site region (E, F).
(TIF)

Figure S3 **CSP profiles where the proton and nitrogen CSPs are combined.**
(TIF)

Figure S4 **Calculated CSP sign differences between various binding modes of fragment 1.** Comparative CSP sign analysis showing the CSP sign differences observed for protein protons (in red spheres) when the catechol orientation is modified. Two different cases are displayed in (A) and (B) showing that the CSP sign differences depend on the relative binding modes of the catechol moieties. Spheres are displayed only if one of the two compared fragment protons exhibits calculated CSPs larger than 0.02 ppm.
(TIF)

Figure S5 **Electronic density observed for the fragments in the crystal structures for compound 1 (A), 2 (B), 3 (C) and 4 (D).**
(TIF)

Figure S6 **Comparative CSP sign analysis for fragment 5 upon binding to PRDX5.** (A) The 3 binding modes of fragment 5 determined by CSP calculation (Figure 3B) are analysed through the comparative CSP sign analysis. CSP sign differences expected between fragment 1 (green) and fragment 5 are displayed (red spheres) for the orientation of fragment 5 in violet (B), in cyan (C) and in orange (D). Best agreement with the experimental CSP sign analysis is observed in the case of the orientation displayed in (C). Spheres are displayed only if one of the two compared fragment protons exhibits calculated CSPs larger than 0.02 ppm.
(TIF)

Table S1 **Data collection and refinement statistics (molecular replacement).**
(DOC)

Table S2 **Reduction of the number of distinguishable ligand poses by the CSP Filter.** The number of clusters in all 200 docked positions of each fragment is compared to the number of clusters in the first 10% of ligand positions selected by the CSP Filter. Two different cluster thresholds of RMSD = 2 Å and RMSD = 1 Å were used.
(DOC)
