# Supplementary figures and images for: Comparing Binding Modes of Analogous Fragments Using NMR in Fragment-Based Drug Design: Application to PRDX5
Source: PLoS One. 2014 Jul 15;9(7):e102300. doi: 10.1371/journal.pone.0102300 (PMC4099364; doi:10.1371/journal.pone.0102300)

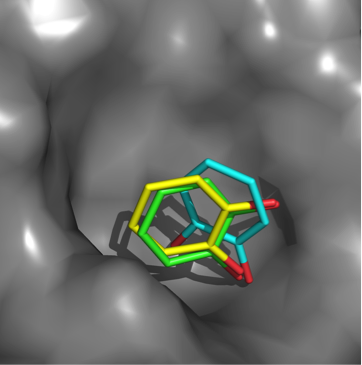

Supplement: Figure S1 — CSP-driven binding mode for fragment 1. The NMR structure is shown in cyan, the X-Ray structure is coloured in yellow, and a docking position close to the X-Ray structure is shown in green. Fragment position was extracted from the solved X-Ray structure and is displayed in the 3MNG protein structure (used for docking). As shown in Figure S1, the binding mode selected by the CSP calculation for compound 1 is quite different form the X-Ray structure (rmsd 2.69 Å). This result arises form the Pscore filter that identifies as the best position the orientation displayed in cyan and not the orientation coloured in green (rmsd 0.36 Å to the X-Ray structure). Here, additional NMR data would be required to select the structure displayed in green. This false positive result highlights limitation of the CSP calculation, similarly to the function scoring issues reported for fragment docking. The limitation of the CSP calculation is likely to increase for cases where experimental CSPs are small and/or measured for a small set of protein residues. For example here, a smaller number of CSPs were used for fragment 1 as compared to the others compounds. (TIF) [file pone.0102300.s001.tif]

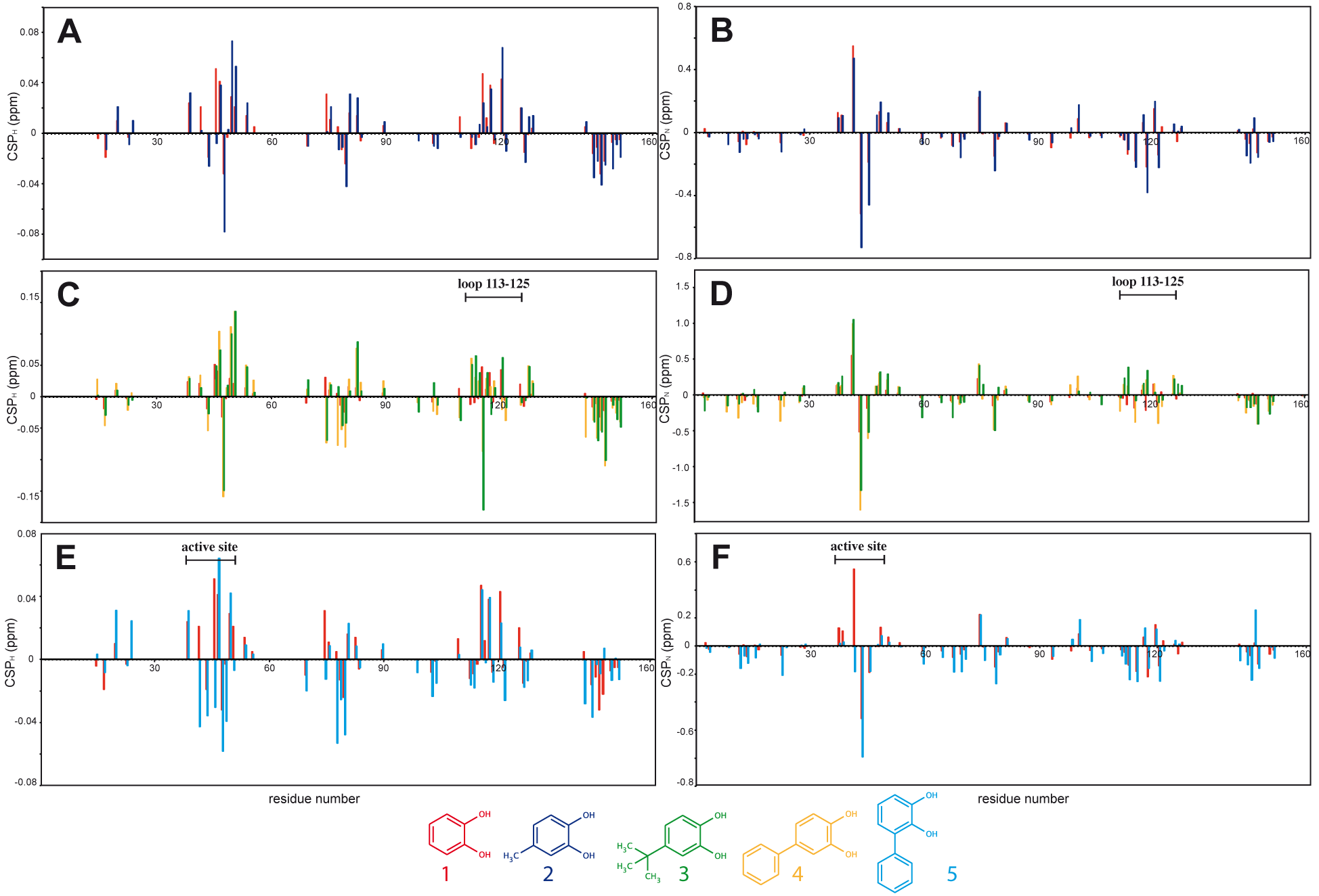

Supplement: Figure S2 — CSP profiles for fragments 1–5, observed for protein protons (left) and protein nitrogen atoms (right). CSP profiles are superimposed for fragments 1 and 2, showing few differences (A, B), fragments 1, 3, 4, showing differences for the loop 113–125 (C, D) and fragments 1 and 5, showing differences in the active site region (E, F). (TIF) [file pone.0102300.s002.tif]

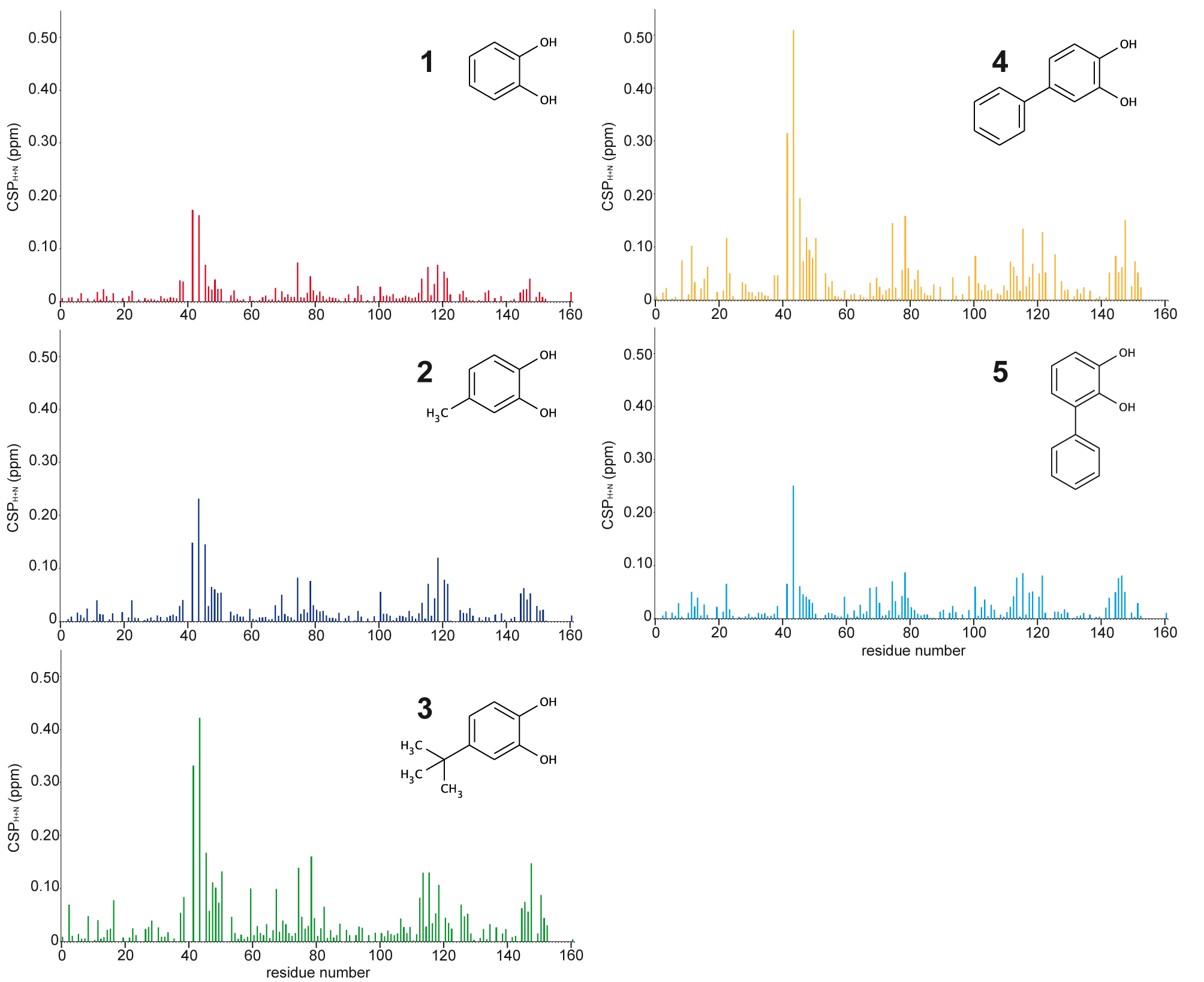

Supplement: Figure S3 — CSP profiles where the proton and nitrogen CSPs are combined. (TIF) [file pone.0102300.s003.tif]

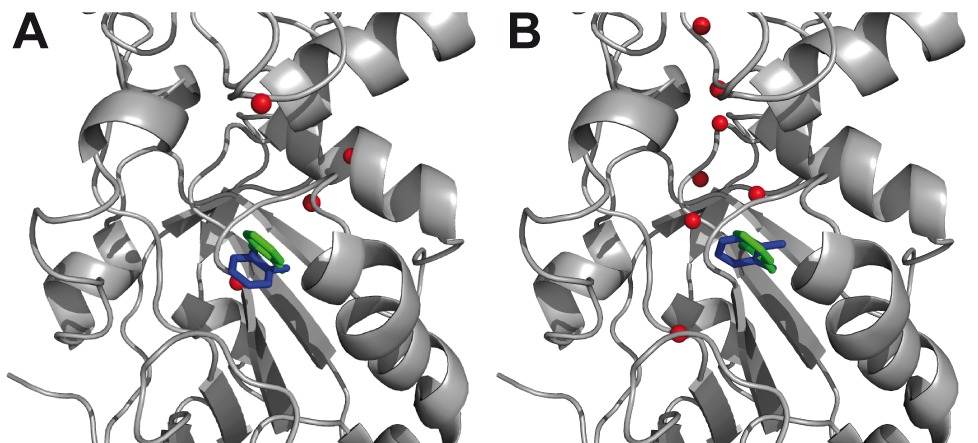

Supplement: Figure S4 — Calculated CSP sign differences between various binding modes of fragment 1. Comparative CSP sign analysis showing the CSP sign differences observed for protein protons (in red spheres) when the catechol orientation is modified. Two different cases are displayed in (A) and (B) showing that the CSP sign differences depend on the relative binding modes of the catechol moieties. Spheres are displayed only if one of the two compared fragment protons exhibits calculated CSPs larger than 0.02 ppm. (TIF) [file pone.0102300.s004.tif]

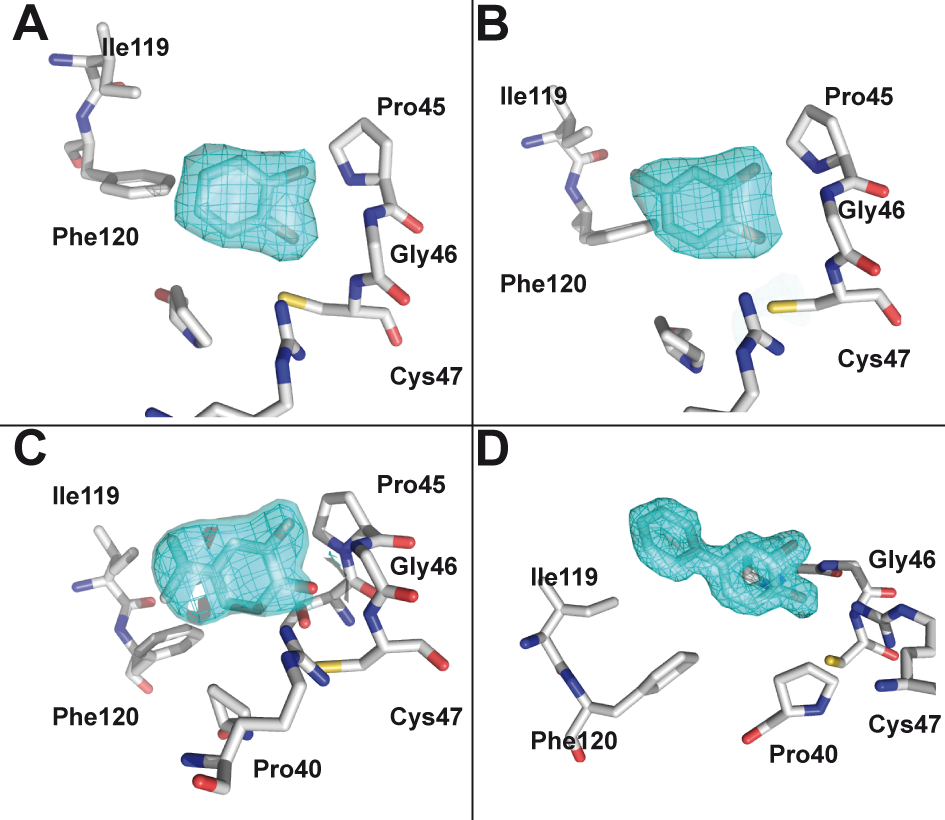

Supplement: Figure S5 — Electronic density observed for the fragments in the crystal structures for compound 1 (A), 2 (B), 3 (C) and 4 (D). (TIF) [file pone.0102300.s005.tif]

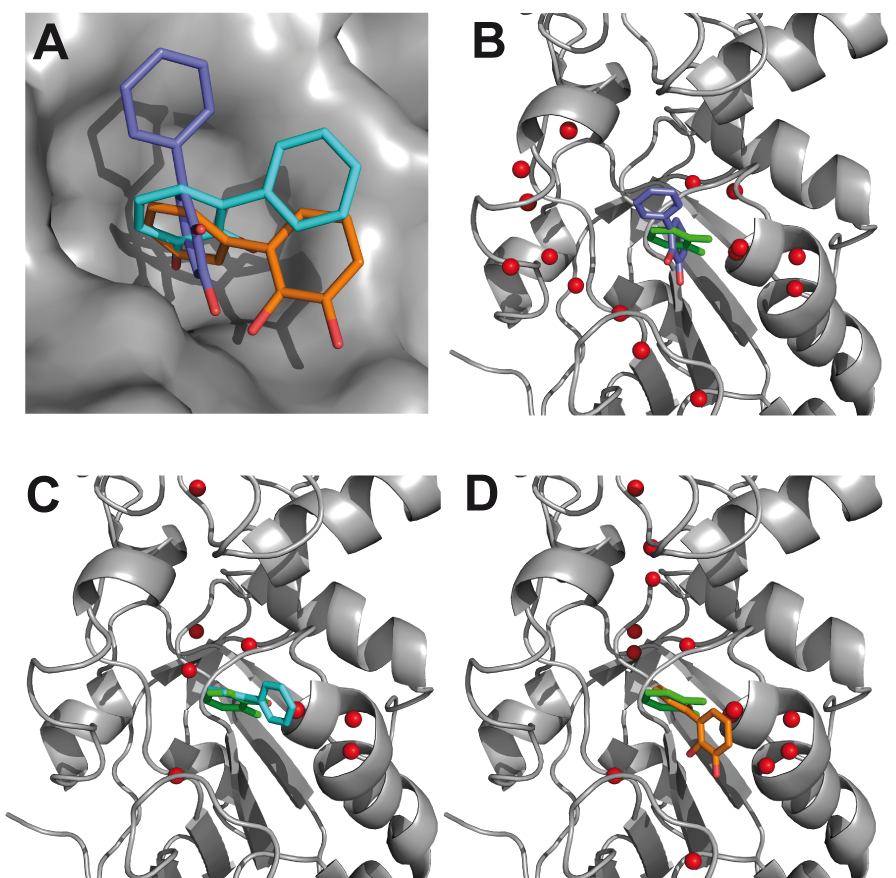

Supplement: Figure S6 — Comparative CSP sign analysis for fragment 5 upon binding to PRDX5. (A) The 3 binding modes of fragment 5 determined by CSP calculation (Figure 3B) are analysed through the comparative CSP sign analysis. CSP sign differences expected between fragment 1 (green) and fragment 5 are displayed (red spheres) for the orientation of fragment 5 in violet (B), in cyan (C) and in orange (D). Best agreement with the experimental CSP sign analysis is observed in the case of the orientation displayed in (C). Spheres are displayed only if one of the two compared fragment protons exhibits calculated CSPs larger than 0.02 ppm. (TIF) [file pone.0102300.s006.tif]
