# Supplementary material for: Comparing Binding Modes of Analogous Fragments Using NMR in Fragment-Based Drug Design: Application to PRDX5
Source: PLoS One. 2014 Jul 15;9(7):e102300. doi: 10.1371/journal.pone.0102300 (PMC4099364; doi:10.1371/journal.pone.0102300)
Supplement: Table S1 — Data collection and refinement statistics (molecular replacement). (DOC) [file pone.0102300.s007.doc]

**Table S1.**

|  | **1** | **2** | **3** | **4** |
| --- | --- | --- | --- | --- |
| **Data collection** | | | | |
| Space group | C222_1_ | C222_1_ | C222_1_ | P2_1_ |
| **Cell dimensions ** | | | | |
| *a*, *b*, *c* (Å) | 79.6, 103.8, 144.9 | 80.1, 101,5, 155,5 | 84.9, 101.5, 148.7 | 66.5, 101.2, 58,7 |
|    γ (°) | 90.0, 90.0, 90.0 | 90.0, 90.0, 90.0 | 90.0, 90.0, 90.0 | 90.0, 90.09, 90.0 |
| Resolution (Å) | 47.6 (2.25)* | 48.9 (2.30)* | 65.1 (1.98) | 44.05 (1.47)* |
| *R*_sym_ or *R*_merge_ | 0.090 (0.323) | 0.057 (0.335) | 0.100 (0.393) | 0.090 (0.471) |
| *I* / *I* | 5.3 (2.1) | 9.1 (1.9) | 6.0 (1.8) | 5.7 (1.6) |
| Completeness (%) | 98.9 (99.6) | 99.7 (99.8) | 99.9 (99.8) | 99.8 (99.6) |
| Redundancy | 4.8 (4.9) | 5.7 (6.0) | 11.3 (11.4) | 4.7 (4.7) |
| **Refinement** | | | | |
| Resolution (Å) | 2.25 | 2.30 | 1.98 | 1.47 |
| No. reflections | 28855 | 26994 | 45081 | 119074 |
| *R*_work_ / *R*_free_ | 17.5/22.6 | 18.7/23.5 | 17.6/21.6 | 16.2/19.2 |
| No. atoms |  |  |  |  |
| Protein | 3580 | 3580 | 3644 | 4881 |
| Ligand | 24 | 27 | 36 | 28 |
| Water | 482 | 453 | 440 | 861 |
| *B*-factors |  |  |  |  |
| Protein | 42.4 | 46.5 | 27.6 | 13.2 |
| Ligand | 44.6 | 45.2 | 44.6 | 19.4 |
| Water | 40.8 | 41.8 | 34.8 | 22.9 |
| **R.m.s. deviations** | | | | |
| Bond lengths (Å) | 0.013 | 0.009 | 0.010 | 0.007 |
| Bond angles (°) | 1.70 | 1.40 | 1.47 | 1.38 |

*Values in parentheses are for highest-resolution shell.
