# Supplementary material for: Comparing Binding Modes of Analogous Fragments Using NMR in Fragment-Based Drug Design: Application to PRDX5
Source: PLoS One. 2014 Jul 15;9(7):e102300. doi: 10.1371/journal.pone.0102300 (PMC4099364; doi:10.1371/journal.pone.0102300)
Supplement: Table S2 — Reduction of the number of distinguishable ligand poses by the CSP Filter. The number of clusters in all 200 docked positions of each fragment is compared to the number of clusters in the first 10% of ligand positions selected by the CSP Filter. Two different cluster thresholds of RMSD = 2 Å and RMSD = 1 Å were used. (DOC) [file pone.0102300.s008.doc]

**Table S2.**

| Fragments | Number of clusters for 200 docked positions | | Number of clusters for 10% of CSP-filtered positions | |
| --- | --- | --- | --- | --- |
|  | 2 Å | 1 Å | 2 Å | 1 Å |
| **1** | 4 | 6 | 1 | 2 |
| **2** | 2 | 8 | 1 | 3 |
| **3** | 10 | 31 | 1 | 4 |
| **4** | 8 | 30 | 1 | 2 |
| **5** | 12 | 30 | 3 | 4 |
